# Effect of Change in the Supplemental Nutrition Assistance Program Guidelines on Vendor Participation and Availability of Fresh Produce

**DOI:** 10.5888/pcd16.190020

**Published:** 2019-08-22

**Authors:** Akiko S. Hosler, Xiao Cong

**Affiliations:** 1 Department of Epidemiology and Biostatistics, University at Albany, Rensselaer, New York

## Abstract

**Introduction:**

In January 2018, new vendor eligibility standards for the Supplemental Nutrition Assistance Program (SNAP) were fully implemented to increase availability of healthy staple and perishable foods. We examined changes in SNAP vendor participation and availability of fresh fruits and vegetables (FFV) both short-term (2015 vs 2018) and long term (2003 vs 2018) in an urban, low-income community.

**Methods:**

We conducted food store assessments from late June through early September of 2003, 2009, 2012, 2015, and 2018 in Albany, New York. SNAP status was assessed by using the US Department of Agriculture’s list of SNAP-authorized stores and in-store verification.

**Results:**

Numbers of SNAP vendors were 77 in 2003, 92 in 2009, 103 in 2012, 115 in 2015, and 109 in 2018. We observed a marginally significant (*P* = .049) short-term (2015, 85.9% vs 2018, 73.9%) decline in SNAP participation among convenience stores but no significant short-term changes in FFV availability among either SNAP or non-SNAP vendors. In long-term (2003 through 2018) trends, we found significant (*P* < .01) increases in SNAP participation among farmers markets and nonprofit organizations. The proportion of SNAP vendors stocking only 1 type of FFV also significantly increased, which was likely related to a consumer trend of favoring bananas as a grab-and-go snack.

**Conclusion:**

Despite the decline of SNAP participation among convenience stores, which primarily came from increased program withdrawals, the new SNAP rule did not substantially alter FFV availability after 6 to 8 months of its full implementation. Long term, policy efforts increased SNAP participation among farmers markets.

**Summary**

**What is already known on this topic?**

Healthy food availability is generally low among SNAP (Supplemental Nutrition Assistance Program) vendors in low-income communities. A new SNAP rule was introduced in January 2018 to increase availability of healthy staple and perishable foods among SNAP vendors, though the rule prompted some concerns, such as decreased SNAP participation among small food stores that do not have resources to meet the new requirements.

**What is added by this report?**

This is one of the first studies to report a change in SNAP vendor participation (ie, a decline in SNAP participation among convenience stores) and its impact on the food environment (ie, no significant change in fresh fruit and vegetable availability) after the implementation of the new SNAP rule in a low-income community. Our study also found important 15-year trends in SNAP vendor participation and fresh fruit and vegetable availability in the same community to provide contextual information.

**What are the implications for public health practice?**

The results of our study suggest that the food environment changes slowly, and some changes can be led by government policy and programs while others can be influenced by secular trends, such as consumer preference. Continuous monitoring and long-term data are important to evaluate food policy and its impact on the food environment.

## Introduction

The Supplemental Nutrition Assistance Program (SNAP) is the largest public food assistance program for low-income individuals and families in the United States and had more than 42.1 million participants in 2017 (1). SNAP benefits, which average $126 per person per month, can be redeemed at any SNAP-authorized food vendor nationwide (1). A common concern regarding SNAP vendors has been low availability of healthy foods (2,3). In low-income communities in particular, SNAP vendors are usually convenience stores and small grocers (4–6), where the availability of fresh fruits and vegetables (FFV) is low (5,7,8).

In response to this concern, the US Department of Agriculture (USDA) published a new SNAP vendor eligibility rule entitled “Enhancing Retailer Standards in the SNAP” in December 2016, and fully implemented it in January 2018. This new rule requires most SNAP vendors to continuously carry 3 stocking units of 3 staple food varieties (types) in each of the 4 staple food categories (meat/poultry/fish, bread/cereal, fruits/vegetables, and dairy), including at least 1 perishable type from at least 2 staple food categories (9).

Before the rule’s implementation, the business sector voiced concern that the new rule would disproportionately burden small food stores, which might lack space or equipment to stock required items, resulting in fewer numbers of SNAP vendors (10). Whether the new rule would reduce SNAP vendors, and how this would influence access to healthy foods in low-income communities have not been fully investigated. Furthermore, the contextual information, such as long-term, secular changes in SNAP vendor participation and healthy food availability are not well understood. The purpose of our study was to investigate changes in SNAP vendor participation and availability of FFV in food stores both short term (2015 vs 2018) and long term (2003 through 2018) in an urban, low-income community.

## Methods

### Study setting

The setting of our study was an urban, mixed land–use area consisting of 49 census block groups in Albany, New York. The study area had a population of approximately 54,500. Our research team focused on this study area as a priority community because of its disadvantaged socioeconomic status, reduced access to healthy foods, and a high need for public health intervention. Population census data for 2010 indicated that 29% of residents had a household income below 100% of the federal poverty line, and 43% belonged to a racial/ethnic minority group (11). Analysis of 2013 American Community Survey data found that nearly all census block groups that formed the study community (47 of 49) fell between the 7th and 10th decile of the Area Deprivation Index state ranking, indicating elevated neighborhood deprivation (12). Furthermore, according to USDA’s Food Access Research Atlas (13), in 2015 the study community encompassed Albany’s entire low food–access areas (ie, low-income census tracts where a substantial share of residents need to travel more than 1 mile to reach the nearest supermarket) (14). For the city as a whole, 31.5% of residents were food insecure (15) and 20.5% received SNAP benefits in 2013–2014 (16). These figures were likely to be higher for the study community because of a higher proportion of low-income residents than the rest of the city.

### Data collection

As part of an ongoing food environment research project, we conducted a baseline food store assessment in 2003, and 4 additional waves of assessments in 2009, 2012, 2015, and 2018 during summer, from late June through early September. Food stores were defined as food retail outlets that stocked any of the following items: loaves of bread, milk, or fruits and vegetables that were fresh, canned, or frozen. Stores that were located inside office buildings with no public access or limited public access were not eligible. By using our established protocol, we compiled multiple government administrative directories of retail stores to identify locations of food stores, then canvassed the study community by car or on foot to verify stores’ eligibility and find stores not on the lists (17).

In each data collection wave, we conducted store assessment at all eligible food stores, including stores that opened after the previous data collection wave. We developed our own food store assessment tool and used it in the baseline assessment in 2003 (18). Subsequently we modified the tool to increase usability and community relevance, renamed it the food retail outlet survey tool, and tested its interrater reliability in 2009 (19). The method of FFV assessment was consistent throughout the study period. When a store stocked fewer than 10 types of FFV, we wrote down the name of each type (eg, tomato, banana, onion). When the store had more than 10 FFV types, we wrote “a large variety” (in 2003) or checked off the box for more than 10 varieties in the survey tool (all other years). We also recorded any noteworthy observations relating to quality and quantity of FFV in the survey’s comment section.

For identification of status as SNAP vendor, we obtained lists of SNAP-authorized stores through special requests to the USDA Food and Nutrition Service Redemption Management Branch (2003 and 2009) and through the USDA Food and Nutrition Service website (2012, 2015, and 2018) (20). We also asked a store employee or manager whether the store took food stamps/Electronic Business Transfer (EBT) (all years), and we visually confirmed the presence of an EBT terminal in the store (2012, 2015, and 2018). Other store characteristics were recorded during the assessment, except for square footage of store buildings, data for which were obtained from the New York State Department of Agriculture’s inspected food retailers’ lists and supplemented by online real property information resources. The University at Albany Institutional Review Board reviewed and approved the study protocols.

### Data analysis

For the SNAP vendor participation analysis, we first examined longitudinal SNAP vendor participation status by data collection cohort. We grouped stores into 4 mutually exclusive categories: 1) continuous SNAP vendor, 2) continuous non-SNAP vendor, 3) changed SNAP status at least once, and 4) no longer a food store. Next, we obtained cross-sectional counts and proportions of SNAP vendors overall and by store characteristics in each data collection wave. The store characteristics we selected were store type (supermarket, ethnic market, convenience store, specialty food store, prepared food store with a grocery section, pharmacy, dollar store, or farmers market), number of cash registers (1, 2 or 3, or ≥4), square footage of the store building (<1,600, 1,600–4,999, or ≥5,000), business hours per week (<98 h or ≥98 h), and ownership type (corporate business, independent business, or nonprofit organization).

For FFV availability analysis, we obtained cross-sectional counts and proportions of stores carrying specific numbers of FFV types (none, 1, 2–4, 5–9, and ≥10) in each data collection year. We excluded an FFV type that had fewer than 3 stocking units to align with the SNAP shelving requirement of at least 3 units or more. We tabulated the results by SNAP and non-SNAP vendors separately. For stores carrying only 1 type of FFV, we identified the one most frequently sold.

Significance of the short-term changes (before new rule implementation in 2015 vs after new rule implementation) was evaluated by the χ^2^ test, and significance of the long-term changes (2003 through 2018) was evaluated by the Cochran-Armitage test of linear trend. Analyses were conducted by using SPSS-PC, version 25 (IBM Corporation) and WINPEPI, version 11.65 (JH Abramson) (21).

## Results

A total of 179 unique stores were in the study area during the study period (Table 1). Among those 179 stores, 31 stores (17.3%) were no longer food stores by 2018, 84 (46.9%) were continuous SNAP vendors, 25 (14.0%) were continuous non-SNAP vendors, and 39 (21.8%) changed SNAP participation status at least once. The 2003 baseline cohort (n = 111) had a similar distribution of categories; 21.6% changed SNAP status at least once.

**Table 1:** Longitudinal SNAP Vendor Participation Status, Albany, New York, 2003–2018^a^

Status in 2018	2003 Cohort (Baseline)	2009 Cohort	2012 Cohort	2015 Cohort	2018 Cohort	Total
Continuous SNAP vendor	55 (49.5)	5 (23.8)	8 (36.4)	9 (69.2)	7 (58.3)	84 (46.9)
Continuous non-SNAP vendor	14 (12.6)	2 (9.5)	2 (9.1)	2 (15.4)	5 (41.7)	25 (14.0)
Changed SNAP status at least once	24 (21.6)	8 (38.1)	6 (27.3)	1 (7.7)	NA	39 (21.8)
No longer a food store	18 (16.2)	6 (28.6)	6 (27.3)	1 (7.7)	NA	31 (17.3)
Total number	111	21	22	13	12	179

Abbreviations: NA, not applicable; SNAP, Supplemental Nutrition Assistance Program.

a Values are number (percentage) unless otherwise indicated.

We assessed 111 food stores in 2003, 124 in 2009, 139 in 2012, 142 in 2015, and 148 in 2018 (Table 2). Numbers of SNAP vendors were 77 in 2003, 92 in 2009, 103 in 2012, 115 in 2015, and 109 in 2018. We saw noticeable variations in SNAP vendor participation by store characteristics. SNAP participation was generally higher among large businesses. Supermarkets, pharmacies, stores with 4 or more cash registers, stores with building size of 5,000 square feet or larger, and stores owned by corporations had a participation rate of 80% or higher in all data collection years. Prepared-food stores with a grocery section were least likely to participate in SNAP, with participation rates that ranged from 0% to 22.2% by data collection year.

**Table 2:** Trends of SNAP Vendor Participation by Store Characteristics, Albany, New York, 2003–2018^a^

Characteristic	2003	2009	2012	2015	2018	*P* Value for Change, 2015–2018	*P* Value for Trend, 2003–2018
**All stores**	77/111 (69.4)	92/124 (74.2)	103/139 (74.1)	115/142 (81.0)	109/148 (73.6)	.14	.24
**Store type**							
Supermarket	4/4 (100)	4/4 (100)	6/6 (100)	6/6 (100)	7/7 (100)	>.99	NA
Ethnic grocery	8/11 (72.7)	10/11 (90.9)	10/14 (71.4)	11/14 (78.6)	13/17 (76.5)	.89	.89
Convenience store	51/72 (70.8)	61/78 (78.2)	67/83 (80.7)	73/85 (85.9)	65/88 (73.9)	.05	.41
Specialty-food store^b^	5/7 (71.4)	3/5 (60.0)	3/5 (60.0)	3/6 (50.0)	2/4 (50.0)	>.99	.40
Prepared-food store^c^	0/4 (0)	1/6 (16.7)	2/9 (22.2)	1/7 (14.3)	1/9 (11.1)	.85	.88
Pharmacy	8/8 (100)	8/9 (88.9)	8/9 (88.9)	9/10 (90.0)	9/10 (90.0)	>.99	.56
Dollar store	1/2 (50.0)	3/5 (60.0)	6/9 (66.7)	8/9 (88.9)	7/8 (87.5)	.93	.09
Farmers market	0/3 (0)	2/6 (33.3)	1/4 (25.0)	4/5 (80.0)	5/5 (100)	.32	.001
**No. of cash registers**							
1	48/73 (65.8)	59/82 (72.0)	71/96 (74.0)	83/104 (79.8)	76/110 (69.1)	.07	.44
2 or 3	20/28 (71.4)	22/29 (75.9)	19/27 (70.4)	18/23 (78.3)	19/24 (79.2)	.94	.52
≥4	9/10 (90.0)	11/13 (84.6)	13/16 (81.3)	14/15 (93.3)	14/14 (100)	.33	.23
**Building square footage**							
<1,600	33/54 (61.1)	40/58 (69.0)	46/66 (69.7)	51/66 (77.3)	45/66 (68.2)	.24	.26
1,600 to 4,999	26/39 (66.7)	34/47 (72.3)	38/52 (73.1)	42/53 (79.2)	42/60 (70.0)	.26	.60
≥5,000	18/18 (100)	18/19 (94.7)	19/21 (90.5)	22/23 (95.7)	22/22 (100)	.32	.87
**Business hours per week**							
<98	25/45 (55.6)	35/58 (60.3)	43/63 (68.3)	43/61 (70.5)	40/61 (65.6)	.56	.17
≥98	52/66 (78.8)	57/66 (86.4)	60/76 (78.9)	72/81 (88.9)	69/87 (79.3)	.09	.85
**Ownership**							
Corporation	19/23 (82.6)	21/24 (87.5)	23/28 (82.1)	24/28 (85.7)	24/28 (85.7)	1.00	.84
Independent business	57/84 (67.9)	68/94 (72.3)	78/106 (73.6)	86/108 (79.6)	79/114 (69.3)	.08	.56
Nonprofit organization	1/4 (25.0)	3/6 (50.0)	2/5 (40.0)	5/6 (83.3)	6/6 (100)	.32	.006

Abbreviation: NA, not applicable; SNAP, Supplemental Nutrition Assistance Program.

a Data shown for years are number of SNAP vendors (numerator) and number of all stores (denominator), with percentage in parentheses.

b Specialty-food stores include meat, sausage, seafood, bakery, cheese, and natural food stores.

c Prepared-food stores that had a grocery section.

In terms of potential effects of the new rule, a decline in overall SNAP vendor participation rate from 2015 to 2018 (81% to 73.6%) was not significant (*P* = .14). However, we saw a marginally significant decline in the SNAP participation rate among convenience stores (85.9% to 73.9%, *P* = .049) during the same time period. Our auxiliary analysis revealed that during the period from 2015 through 2018, the gain of SNAP convenience stores by participation of new stores (n = 4) and existing stores (n = 3) was smaller compared with earlier periods (Table 3). Additionally, 11 convenience stores withdrew from SNAP between 2015 and 2018. In comparison, only 1 convenience store withdrew between 2012 and 2015. As for long-term trends of SNAP vendor participation, significant linear increases were observed among farmers markets (0 in 2003 to 100% in 2018, *P* = .001) and stores run by nonprofit organizations (25.0% in 2003 to 100% in 2018, *P* = .006).

**Table 3:** SNAP Vendor Gain and Loss Among Convenience Stores, Albany, New York, 2003–2018^a^

Change	2003–2009	2009–2012	2012–2015	2015–2018
Gain by participation of new stores	6	8	6	4
Gain by participation of existing stores (non-SNAP to SNAP)	9	5	6	3
Total gain	15	13	12	7
Loss by store closing	3	2	5	4
Loss by withdrawal (SNAP to non-SNAP)	2	5	1	11
Total loss	5	7	6	15
Net gain or loss	10	6	6	−8

Abbreviation: SNAP, Supplemental Nutrition Assistance Program.

a Data are number of stores.

No significant changes occurred in FFV availability before and after the new rule implementation among either SNAP or non-SNAP vendors (Table 4). Proportions of SNAP stores with only 1 type of FFV increased from 10.4% in 2015 to 18.3% in 2018, but the difference was not significant (*P* = .09). In 2015, two-thirds of SNAP vendors (66.1%) already stocked at least 1 type of FFV, and this proportion changed little in 2018 (to 67.0%). In terms of long-term trends, we observed a significant linear trend of decline in SNAP vendors without FFV (55.8% in 2003 to 33.0% in 2018, *P* = .002), and a significant linear trend of increase in SNAP vendors stocking 1 type of FFV (1.3% in 2003 to 18.3% in 2018, *P* = .001). Among SNAP vendors stocking only one FFV type, banana was the one most frequently sold — the grab-and-go snack — in all data collection years.

**Table 4:** Trends of Fresh Fruit and Vegetable Availability Among SNAP and Non-SNAP Vendors, Albany, New York, 2003–2018^a^

Fresh Fruit and Vegetable Availability^b^	2003	2009	2012	2015	2018	*P* Value for Change, 2015–2018	*P* Value for Trend, 2003–2018
**SNAP vendors, total no. types**	77	92	103	115	109	NA	NA
None	43 (55.8)	37 (40.2)	40 (38.8)	39 (33.9)	36 (33.0)	.89	.002
1	1 (1.3)	10 (10.9)	11 (10.7)	12 (10.4)	20 (18.3)	.09	.001
2–4	12 (15.6)	18 (19.5)	21 (20.4)	27 (23.5)	23 (21.1)	.67	.28
5–9	8 (10.4)	11 (12.0)	15 (14.6)	15 (13.0)	7 (6.4)	.10	.42
≥10	13 (16.9)	16 (17.4)	16 (15.5)	22 (19.1)	23 (21.1)	.71	.39
**Non–SNAP vendors, total no. types**	34	32	36	27	39	NA	NA
None	23 (67.6)	18 (56.3)	21 (58.3)	17 (63.0)	27 (69.2)	.60	.67
1	5 (14.7)	3 (9.4)	2 (5.6)	2 (7.4)	2 (5.1)	.70	.15
2–4	2 (5.9)	5 (15.6)	5 (13.9)	2 (7.4)	6 (15.4)	.33	.47
5–9	0	1 (3.1)	2 (5.6)	3 (11.1)	2 (5.1)	.37	.15
≥10	4 (11.8)	5 (15.6)	6 (16.7)	3 (11.1)	2 (5.1)	.37	.28

Abbreviations: NA, not applicable; SNAP, Supplemental Nutrition Assistance Program.

a Values are number (percentage) unless otherwise indicated.

b The most frequently sold fruit or vegetable type was banana in all years: Numbers of stores stocking bananas were 1 store (100%) in 2003, 5 stores (50%) in 2009, 8 stores (73%) in 2012, 9 stores (60%) in 2015, and 13 stores (59%) in 2018.

We examined the spatial distribution of SNAP vendors and farmers markets over time in relation to population density (Figure). In 2003, SNAP vendors tended to cluster in the most densely populated sections of the study community (ie, population density ≥15,000 people per square mile), and fewer than 50% of SNAP vendors stocked at least 1 type of FFV. There were also several pockets where SNAP vendors with FFV were lacking. Gradually, the number of SNAP vendors with at least 1 type of FFV increased, and they also spread more evenly across the study community. By 2018, the study community was fairly saturated with SNAP vendors with at least 1 type of FFV, and even the least populated sections (ie, population density <5,000 people per square mile) had a SNAP vendor with FFV or a farmers market.

**Figure:**
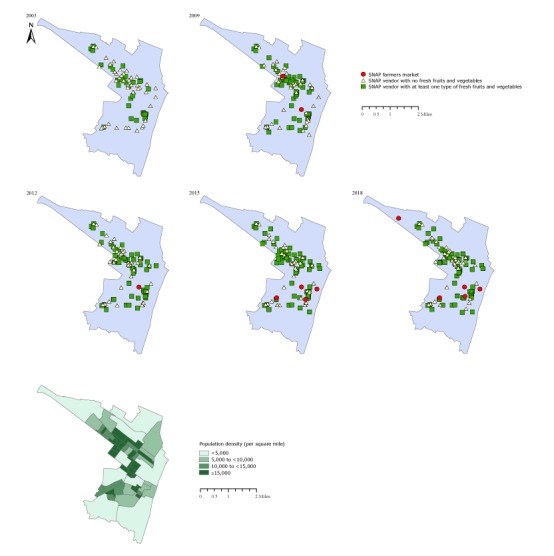
Spatial distributions in 2003, 2009, 2012, 2015, and 2018 of SNAP farmers markets and SNAP vendors in Albany, New York, with no fresh fruits or vegetables and those with at least 1 type of fresh fruit or vegetable, and population density of Albany, New York. Source: 2006-2010 American Community Survey 5-year estimates for Albany, New York (22).

## Discussion

Convenience stores, which were the most common SNAP vendors in our study community, were the only type of vendors that experienced a significant decline in SNAP participation from 2015 to 2018. We found a more than 10-fold increase in SNAP withdrawals among convenience stores in this time period compared with the period from 2012 through 2015. Although moving in and out of SNAP was not uncommon, the sudden increase in program withdrawals for the 2015–2018 period suggested an effect of the new rule.

We observed a small, short-term increase in the proportion of SNAP vendors that stocked 1 FFV, but it was not significant. The new rule defines perishable staple food varieties as “either frozen or fresh staple food items or fresh, unrefrigerated or refrigerated staple food items that would spoil or suffer significant deterioration in quality within 2–3 weeks at room temperature” (23). Therefore, a large number of common food items, such as fresh milk (dairy category), bread (bread/cereal category), and chicken eggs (meat/poultry/fish category), as well as frozen fruits and vegetables qualify for this definition. We focused on FFV because they were the least available perishable healthy food in our study community, but the new rule has no specific requirements or incentives to choose FFV for perishable food.

In terms of long-term trends, significant increases in SNAP participation rates among farmers markets and nonprofit organizations deserve further discussion. (Note that all farmers markets in the study area were operated by nonprofit organizations).

From 2003 to 2012, participation in SNAP among farmers markets remained low in our study community, in the range of 0 to 33%. Literature indicated that this low rate was largely due to barriers to adopting a wireless EBT system (24). In New York State, migration from paper vouchers to EBT was completed in June 2009, but there was no federal requirement that wireless devices be provided free of charge to SNAP vendors. Subsequently the USDA began a series of interventions to expand the use of wireless point-of-sales devices in farmers markets, such as initiating a grant program in 2013 (25) and sending letters to state agencies to enhance the availability of no-cost wireless devices in 2016 (26). The technology sector also responded by creating inexpensive, fast, and easy-to-operate wireless systems and applications designed for farmers markets. Our study confirmed that by 2018, all farmers markets in our study area had a wireless EBT device.

In addition, in 2012 New York State launched the “FreshConnect” SNAP client incentive program, in which a $2.00 coupon was provided for every $5.00 of SNAP benefit used at participating farmers markets (27). By 2018, all farmers markets in our study community became FreshConnect markets. This long-term trend of expanding SNAP participation among farmers markets also contributed to filling spatial gaps of SNAP FFV vendor coverage in our study community.

Another important long-term trend was the significant increase in the number of SNAP vendors stocking only 1 type of FFV, especially bananas. Our earlier study using 2003–2012 data showed that the “fruit for snack” marketing strategy was bourgeoning in our study community (28). In 2012, we observed that many convenience stores sold bananas by the piece and displayed them near the cash register or on a sandwich counter as a grab-and-go item (28). This marketing strategy remained popular in 2015 and 2018. A continuous high consumer demand for bananas (29) seems to be responsible for converting some no-FFV stores to 1-FFV stores. In addition, bananas are not locally grown and thus cannot be sold at farmers markets. This may also create a competitive advantage for convenience stores to sell this popular produce item.

From the public health policy perspective, the long-term trends of increased proportions of SNAP farmers markets and stores that offer 1 type of FFV are welcome changes, but their effects on community health may be limited. All but 1 farmers markets in our study community were seasonal, and their hours of business during the season were short, with a range of 3 to 8 hours per week. The proportion of SNAP vendors stocking 5 or more types of FFV remained relatively unchanged in the 15-year study period. To increase purchasing of FFV among SNAP clients, more year-round SNAP vendors with greater varieties and quantity of FFV are needed (30). The new SNAP rule is designed to implement the minimum standards of the healthfulness of food inventory, but the rule does not include incentives to stock desirable types of healthy foods that most SNAP clients need.

The business sector predicted the decline in number of SNAP convenience stores, which was related primarily to increased program withdrawals (10). The reduced numbers of SNAP convenience stores could negatively affect access to SNAP in economically disadvantaged communities. We did not investigate whether the increase in program withdrawals was caused by the stores’ inability to meet the new eligibility requirements. Qualitative studies of store owners are needed to fill this information gap.

Our study had limitations. The timing of data collection was dictated by our predetermined research schedule (ie, every 3 years during summer). The postintervention data collection point may have been early to observe widespread effect of the new SNAP rule, and continuous monitoring is necessary. Our study’s findings may be unique and not readily generalizable to other low-income urban communities. Strengths of this study include its 15-year longitudinal design, which allowed us to assess long-term secular trends; accurate identification of store locations and study eligibility through cross-referencing multiple administrative store lists; community canvassing and in-store verification; and the use of a reliability-tested tool for food store assessment.

As SNAP’s mission has expanded from temporary relief of hunger and food insecurity to assuring the dietary quality of its recipients, the need to study the program from multiple perspectives and levels of influences has grown. As for a future research direction, continuous monitoring and evaluation of SNAP vendor participation and associated changes in the comprehensive food environment are needed. Underserved urban and rural communities should be for the focus of this effort. We also suggest more studies to link changes in SNAP vendor participation and healthy food availability to changes in food purchasing patterns, dietary behavior, and health status of SNAP clients.

Although we observed a decline in SNAP participation among convenience stores, the new SNAP rule did not substantially alter FFV availability 6 to 8 months after its full implementation. Continuous monitoring of SNAP vendors and follow-up of SNAP clients are necessary to evaluate effectiveness of the new rule. Long term, policy efforts increased SNAP participation among farmers markets, and there was a likely connection between a consumer trend of favoring bananas as a grab-and-go snack and the increase of SNAP vendors stocking only 1 type of FFV.
